# Germline cells in ovarian surface epithelium of mammalians: a promising notion

**DOI:** 10.1186/1477-7827-10-112

**Published:** 2012-12-17

**Authors:** Onder Celik, Ebru Celik, Ilgin Turkcuoglu, Ercan Yilmaz, Yavuz Simsek, Bulent Tiras

**Affiliations:** 1 Department of Obstetrics and Gynecology, Inonu University, Medical Faculty, Malatya, Turkey; 2 Department of Obstetric and Gynecology, Acibadem University, School of Medicine, Istanbul, Turkey

**Keywords:** Human, Ovarian surface epithelium, Stem cells, Germ line cells, Pluripotency, Oocyte-like cells

## Abstract

It is a long held doctrine in reproductive biology that women are born with a finite number of oocytes and there is no oogenesis during the postnatal period. However, recent evidence challenges this by showing the presence of germ line stem cells in the human ovarian surface epithelium (OSE), which can serve as a source of germ cells, and differentiate into oocyte like structures. Postnatal renewal of oocytes may have enormous therapeutic potential especially in women facing the risk of premature ovarian failure idiopathically or iatrogenically after exposure to gonadotoxic chemotherapy and radiation for cancer therapy.

This article reviews current knowledge on germ line stem cells in human OSE.

## Background

Ovarian surface epithelium (OSE) cells differentiate from peritoneal mesothelial cells through their transformation from mesenchymal into epithelial cells [1]. The function of healthy OSE is regeneration and repeated proliferation of cells after ovulation during the reproductive period. Since apparent paucity of significant functions, OSE has received less attention compared to other components of ovarian tissue. The role of OSE in the pathogenesis of epithelial ovarian carcinoma is well established; however, its role in human oogenesis has remained controversial [2-4]. It has been reported that OSE is the source, at least partially, of granulosa cells in the human, and expresses stem cell markers of both epithelial and mesenchymal origin [3,5].

In 1917, Kingery [6] did explain the concept of follicular regeneration from the surface epithelium of postnatal mouse ovary first before mentioning Simkins [7]. In their pioneering work Simkins et al. described the natural role of OSE as supplying germ cells during the entire fetal period [7]. The role of human OSE as a source of oocytes during intrauterine life has been confirmed by scanning and transmission electron microscopy [8]. Recently oogonial stem cells have successfully been isolated from ovaries of neonate and adult mice as well as from human ovaries [9,10]. However, these studies have received some criticism. Byskov et al. later demonstrated that bromodeoxyuridine (BrdU) negative oocytes could be present in female mice after injection of BrDU [11]. Additionally, statistical and mathematical studies involving ovarian reserve found no evidence to support the occurrence of neoogenesis in adult mammals [12,13]. Regardless, OSE is physiologically much more complex than that would be predicted from its inconspicuous appearance. The present review summarizes current knowledge on OSE as a source of neo-oogenesis.

### Ovarian surface epithelium as a regenerative layer of germinal epithelium

A surprising phenomenon described by Johnson et al. who has demonstrated that the mouse ovary is capable of producing new oocytes and follicles from germ line stem cells (GSC) present in OSE after depletion of ovarian cells by chemotherapy [9]. Moreover, they also showed the presence of presumptive mitotically active GSC, expressing VASA protein specific to germ cells, in or close to OSE from juvenile and adult mice. Concerning the origin of new germ cells in mammalian species and humans, the same group proposed that germ line stem cells could be present within the bone marrow and peripheral blood [10]. The follicles containing immature and mature bona fide oocytes, expressing germ cell and oocyte-specific markers, were shown to be formed after bone marrow transplantation irrespective of BMT interval in adult mice treated with chemotherapy. This study has been widely disputed and, several researchers have challenged their findings. A recent study by Santiquet et al. found that new oocytes could be produced neither after BMT nor grafted bovine embryonic ovarian cortex in PU.1 mice and severe combined immunodeficiency (SCID) mice, treated with chemotherapeutic agents [14]. However, the fertility of SCID mice, exposed to chemotherapeutic agents, has been found to be improved after bone marrow transplantation. Thus, the authors have suggested that, despite chemotherapeutic agents, sparing follicles could be restored of self-tolerance of ovarian antigens; thereby, BM cells may have beneficial effects on ovarian physiology.

One morphological study, supporting oocyte renewal, conducted by Kerr et al. reported that despite a rapid decline in the number of primordial follicles per ovary, as measured by unbiased stereological methods, during the first week of postnatal life, the primordial follicle count remained constant and stable during the period between the 7th and 100th days of life in C57BL/6 mouse [15]. Further, they have also demonstrated the presence of dividing cells in OSE by using antibodies against proliferating cell nuclear antigen and germ cell nuclear antigen in prepubertal mouse.

The concept of follicular renewal in postnatal mouse ovary triggered a significant deal of debate. Eggan et al. failed to demonstrate production of any mature oocytes derived from blood born progenitor germ cells following bone marrow transplantation in parabiotic model and also indicated that a few oocytes could rescue from chemotherapeutic agents [16]. In line with previous report, Liu et al. in 2007 reported a conflicting result that either expression of SCP3 marker for meiosis or oogenesis-associated mRNAs for SPO11, PRDM9, SCP1, TERT and NOBOX could not be detected in adult human ovary [17]. Nevertheless, the authors concluded that the presence of a few numbers of cells with mRNA in human ovaries could not be excluded in the study. The result of that study conducted by Liu et al. was in accordance with a subsequent study using histology and immunohistochemical staining for oogonia cell specific markers (C-KIT, SSEA4, NANOG and OCT4 and the testis tumor related MAGE-A4) on the cortex and medulla of young female ovary, from fetal period to adulthood [17,18]. That study has indicated that there were no oogonia stained with pluripotent immunohistochemical markers after the age of 2 years. The authors have doubted that these findings, however, do not rule out either the possibility of OSE cells de novo transformation into multipotent stem-like cells in postnatal human ovary. Latterly, Kerr et al., using same unbiased, assumption free fractionator method, assessed more ovaries to enhance their previous findings [19]. In that study, 97 ovaries were obtained from mice aged 3–300 days old that were exposed to gonodatoxic agents, or whole-body ionizing radiation. The observations revealed that these ovaries were germ cell nuclear antigen negative and no evidence of regeneration of primordial follicle numbers after either expose to chemotherapeutic agents or ionizing radiation. Moreover, morphological and immunochemical analysis was confirmed by stereological analysis of treated ovaries. These recent observations reported by Kerr et al. support the belief that a finite number of oocytes are formed during fetal period and that no new oocytes are generated after the pool of primordial follicles is established [19].

### Expression of stem cell and germ cell markers in OSE

Observations in adult human ovaries raised a question whether OSE cells express the specific markers for stem and germ cells. Telomerase is a ribonucleoprotein that synthesizes telomere repeats to chromosome ends. It is involved in maintaining telomere length in germ line tissues, which plays an essential role in replicative life span [20]. Wright et al. detected telomerase activity in germ line tissue and stem and cancer cells from fetal, neonatal, and adult testes and ovaries [21]. Subsequently, Kinugawa et al. found that telomerase activity is also present in normal ovaries, but it was decreased with age [22]. Their sample included five women with premature ovarian failure (POF); two of who were categorized as having follicular dysfunction by the virtue of having a high number of primordial follicles present in their ovaries (mean 11.8 follicles per medium power field) while remaining three women had genuine follicle depletion (0.03 follicles per medium power field). Further, POF and postmenopausal women showed either high or low telomerase activity in the ovarian surface cell layer in the POF group, but showed high telomerase activity in postmenopausal group just after scrapping of OSE.

Parrott et al. has shown that human and bovine OSE cells express high levels of c-kit receptor and kit-ligand/stem cell factor (SCF) proteins *in vitro*, and SCF gene expression is significantly increased in cultured OSE cells [23]. A study on gene expression profiling of OSE from women with healthy ovaries or with serous papillary ovarian adenocarcinoma reported that while normal OSE expressed genes leading to adult stem cell maintenance and pluripotency at a high rate, OSE cells from adenocarcinoma samples expressed these genes at very low levels or did not at all [24]. Recent study noted that human epithelial stem-like cells are located in the distal fimbriated part of fallopian tubes; further, these cells are able to express stem-like markers, which may play a role in the initiation of serous tumors in ovary [25,26].

Vrant-Klun et al. reported the presence of primitive oocyte like cells, which expressed specific markers for pluripotency, in OSE from a woman with ovarian carcinoma [27]. This observation led to the hypothesis that stem cells located in OSE may have, in fact, pluripotent (or totipotent?) capacity and can give rise to formation of new oocytes. Parallel to these studies, Bukovsky et al. has shown that OSE cells could be the bipotent source for germ and granulosa cells [28,29].

The *in vivo* observations in adult human ovaries indicated that some OSE stem cells were able to convert into germ cells by asymmetric division [30-33]. Some experimental evidence has been indicated by Bukovsky et al. confirmed the existence of oocyte-like cells in cultured OSE cells, which was scrapped from postmenopausal women [34,35]. Immunohistochemistry analysis indicated that the oocytes derived from OSE expressed VASA and deleted azoospermia-like (DAZL) [36].

Later on Zhang et al. confirmed aforementioned observations [37]. The authors used an enhanced green fluorescent protein transgenic model to evaluate the expression of stem and germ cell markers in adult murine ovaries. They were able to demonstrate the presence of pluripotent surface markers (OCT3/4, MVH, SCF-R, and SSEA-1) as well as meiotic markers (DMC1 and SCP3) in specific cells collected from the periphery of adult murine ovary. Importantly, these cells were distinct from ovarian follicles. Based on their observations, Zhang et al. suggested that these cells maintain a mixed cluster of committed stem cells and also a transitional stage of GSC that may be capable of proliferation and differentiation [37]. Following this study on mice, Vrant-Klun et al. demonstrated evidence of presence of putative stem cells (PSCs), which expressed characteristic markers for pluripotency, in the OSE layer of postmenopausal women and women with POF [38]. The authors also detected a pluripotent pattern of gene transcription in OSE of adult human by reverse transcription-polymerase chain reaction (RT-PCR) analysis. Another study conducted by the same group has provided further insight into the presence of germ stem cells in the OSE of postmenopausal women [39]. In this study, rare putative stem cells with germline characteristics were isolated from OSE of postmenopausal human ovaries devoid of oocytes.

A recent study from our groups used a porcine small intestine submucosa (SIS) patch to repair surgically induced damage to ovarian surface in a rabbit model [40]. We were able to demonstrate development of follicular structures around and in the SIS graft starting from the 28th week post surgery. In addition, noticeable PCNA staining was found in oocytes arrested in the meitotic phase.

A recent study by Parte et al. involving OSE from postmenopausal women and other mammalian species provided further support for the presence of PSCs [41]. They found small round PSCs, which clearly stained by DAPI, spontaneously increased in size and differentiated into oocyte like structures, and these oocyte like structures were surrounded by zona pellucida-like and blastocyst like structures in OSE cultures. In addition to the expression specific markers for pluripotency by the oocyte-like cells obtained post-culture, pluripotent gene transcripts were also detected by RT-PCR in OSE scrapings from humans and other mammalian species. Further evidence of the presence of PSC in OSE was provided by another study involving women with POF. OSE cells obtained by scrapping ovaries devoid of oocytes were cultured in a medium containing follicular fluid [42]. Primitive oocyte-like cells positively stained for alkaline phosphatase and expressed markers of pluripotency (SOX-2 and SSEA-4) as well as the typical genes (OCT4A, SOX-2, NANOG, NANOS, STELLA, CD9, LIN28, KLF4, GDF3, and MYC) for pluripotent stem cells. In view of their findings, the investigators concluded that the PSCs exist in OSE of women with POF.

It becomes relevant to refer the last published study here. Bhartiya et al. showed that the gonadotropin-induced pluripotent very small embryonic stem cells (VSELs) (increased expression of Oct-4A, Nanog) underwent proliferation (increased PCNA staining and Oct-4A expression) and differentiation (increased expression of stella, fragilis, total Oct-4, Vasa and MVH) [43]. Further, the differentiating oocyte undergoes to meiosis and exhibits germ cell markers like Stra-8, Scp3 and Dmc1; besides the developing oocytes surrounded by granulosa like cells assemble primordial follicule *in vitro*. The study demonstrated that upregulation of follicular stimulating hormone receptor through gonadotropin administration to adult mouse leads to increased pluripotent stem cell activity in the OSE, related to meiosis and also exhibits several germ cell markers *in vitro*. In view of these findings, that study proposed that epithelial mesenchymal transition gives rise to granulosa like cells whereas VSELs differentiate to form oocytes.

At this juncture, it is crucial to consolidate the various studies, thus a clear understanding emerges. Table 1 is a list of different studies on ovarian surface epithelium.

**Table 1:** Pivotal observations advanced in support of presence of stem cells in ovarian surface epithelium of mammalian species

**Published studies**	**Study observations**	**Interpretation of published data**
Johnson et al., (2004) [9]	Highly expression of early meiotic markers in surface epithelium of postnatal ovary. Formation of chimeric follicles after transplantation of wild type ovarian tissue onto ovary of GFP expressing transgenic mice.	The regeneration of de novo new primordial follicle suggest that oocytes may arise from a rare uncharacterized population of cells, which are present in BM and peripheral circulation
Johnson et al. (2005) [10]	Bone marrow serves as a source of germ stem cells in adult mammalian, which can be transported by peripheral blood to the ovaries.	Induction of oocyte atresia by gonadotoxic agents possibly mobilized VSELs from BM to enter peripheral circulation
Bukovsky et al. (2004, 2005, 2009, 2011, 2012) [29,30,33-35]	Putative germ cells within the OSE of postnatal ovary differentiate from mesenchymal progenitors in the ovarian tunica albuginea. Large oocyte-like cells expressing zona pellucida proteins are formed in set up cell cultures from scrapped OSE. Reproducible, oocyte-like cells from OSE of ovaries subjected to ovotoxic agents.	These results are in agreement with previous data that stem cells in OSE are able to produce oocyte-like structures [38,39]
Virant-Klun et al. (2008, 2008, 2011) [38,39,42]	VSEL in OSE able to generate oocyte-like cells *in vitro*. These cells expressed pluripotent specific markers. Oocytes derived from VSEL cells de novo underwent parthenogenetic activation to produce blastocyst-like cells with neuronal phenotype and embryoid bodies. These structures expressed pluripotent specific markers Oct-4, Oct-4 A, Nanog, Sox-2, and TERT.	Presence of rare putative stem cells with germline characteristics in the OSE of postmenopausal women accorded the initial finding of existence of GSC in adult mouse ovary [9].
Zhang et al. (2008) [37]	Germ cell markers (OCT-3/4, MVH, SCF-R and SSEA-1) and meiotic markers (DMC1 and SCP3) within specific cells aggregated from the periphery of adult murine ovaries.	Mixed cluster of committed stem cells and also a transitional stage of GSC that may retain the capacity of proliferation and differentiation.
Niikura et al. (2009) [44]	Aged mouse ovaries are able to form premeiotic germ cells (high expression of Stra8 and Daz1), when transfer into a young ovarian environment, they can differentiate into oocytes (increased expression of Oct4-GFP, c-kit, Mvh and SSEA-1).	The concept of rapid decline in the number of follicles being mainly due to decreased oocyte renewal rather than to an accelerated loss.
Zou et al. (2009) [45]	MVH positive FGSCs isolated from adult mice ovaries and retained *in vitro* for months. These cells after transplantation into atretic ovaries of recipient mice by gonadotoxic agents produced new follicle, which fertilized and gave rise to offspring carrying the same gene as their mothers.	This study purified the initial cells expressing germ cell markers, but not early stem cell markers.
Parte et al. (2011) [41]	Two different populations of putative stem cells detected in scrapped OSE of postnatal mammalian ovary, namely VSELs and slightly larger ovarian stem cells termed as OGSCs. VSELs expressed nuclear Oct-4 but the OGSCs exhibited cytoplasmic Oct-4. Pluripotent specific markers were detected in human and sheep OSE. After 3 weeks of OSE cultures, oocyte-like cells expressing c-kit, DAZL, VASA and ZP4 emerged	VSELs are totipotent to pluripotent in nature and form OGSCs, which are able to further differentiate into oocyte-like and neuronal-like structures.
Santiquet et al. (2012) [11]	Chemotherapy sterilized SCID mice could not generate new oocytes after BMT but fertility of mice has improved.	Despite ovarian atresia by chemotherapeutics, sparing follicles could be restored of self-tolerance of ovarian antigens; thereby, BM cells possibly initiate an endocrine/paracrine signal that improves the functionality of ovarian niche.
Bhartiya et al. (2012) [43,46]	Gonadotropin induced pluripotent VSELs underwent proliferation and differentiation (increased expression of stella, fragilis, total Oct-4, Vasa and MVH). Differentiating oocyte undergoes to meiosis and exhibits germ cell markers like Stra-8, Scp3 and Dmc1.	Close association of developing oocytes with mesenchymal cells *in vitro*, which is produced by epithelial to mesenchymal transition of the OSE suggested that new oocytes surrounded by granulosa-like cells assemble as primordial follicles in the OSE of adult ovary.
White et al. (2012) [47]	Purified rare mitotically active cells features in both mouse and human ovaries, generated oocytes (diameter of 35 – 50 micron) *per se* and entered into meiotic division. Injection of GFP transfected human OSCs into the human ovarian cortical tissue strips resulted in generation of GFP expressing follicles one-two weeks after xenografting these strips into diabetic SCID mice. Successfully purified rare cell surface DDX4 positive OGCs from cortical tissue of reproductive aged adult human ovaries.	The study showed that rare cells (expression of cell-surface of DDX4) exist in the OSCs of adult human. Production of chimeric follicle *in vitro* and more importantly *in vivo* when injected into cortical tissue strips from human ovaries.
Hayashi et al. (2012) [48]	After transplantation of both female PGCs and embryonic gonadal somatic cells under ovarian bursa or kidney capsules of recipient mice, induced-ESCs transformed into PGCLCs, which contributes to oocyte-like cells in reconstituted ovaries. These cells matured into fully functional germinal vesicle stage, including multiple layers of granulosa and theca cells *in vivo*.	This study demonstrated that possibility of reconstitute female germline development *in vitro*, not only in mice, but also in humans.

### Very small embryonic like putative stem cells in mature mammalian ovary

Previous reports have proposed that the ovary of adult mammalian consists of two different populations of stem cells, termed as VSELs, and slightly larger ovarian germ stem cells (OGSCs) [41]. Ratajczak et al. isolated small stem cells with a diameter of 2–4 micrometers from murine bone marrow. These cells expressed characteristic markers for embryonic, epiblast stem and primordial germ cells (PGCs) [49]. Ratajczak et al. suggested VSELs were pluripotent stem cells, which were deposited during gastrulation and organogenesis in the mammalian embryo [49]. It has been shown that these cells could differentiate into all three-germ-layer cells in *in vitro* culture [50]. VSELs also expressed several characteristic markers for PGCs (fetal-type alkaline phosphatase (AP), octamer-binding transcription factor 4 (Oct-4), SSEA-1, CXCR4, Mvh, Stella, Fragilis, Nobox, Hdac6). These findings may also suggest a possible close association of those cells with epiblast-derived PGCs. They concluded that, VSELs in adult organs declines with age, and their ability to form spheres containing PGS decreases with time. These observations could explain more efficient regeneration in younger adults compared to older ones.

These observations directed attention towards the possible existence of VSELs in human OSE. Virant-Klun et al. isolated VSELs from OSE obtained from 21 postmenopausal women whose ovaries contained no follicles [39]. Fascinatingly, VSELs were transformed into oocyte-like cells and parthenogenetic blastocyst-like structures *in vitro* culture. More importantly, these oocyte-like cells exhibited positive staining for some pluripotent embryonic stem cell markers such as SSEA-4, Oct-4, Sox-2 and NANOG. Recently, these reported findings have been confirmed by Parte et al. [41]. The presence of VSELs with cytoplasmic OCT-4 in ovaries, which can be isolated by scrapping of OSE spontaneously differentiated into oocyte-like structures *in vitro*. Additionally, this group has also shown slightly larger cells with cytoplasmic OCT-4 that were defined as OGSCs. The presence of two different populations of stem cells was confirmed in adult mouse ovaries by using both immune-localization and quantitative polymerase chain reaction analysis [46].

**Figure 1:**
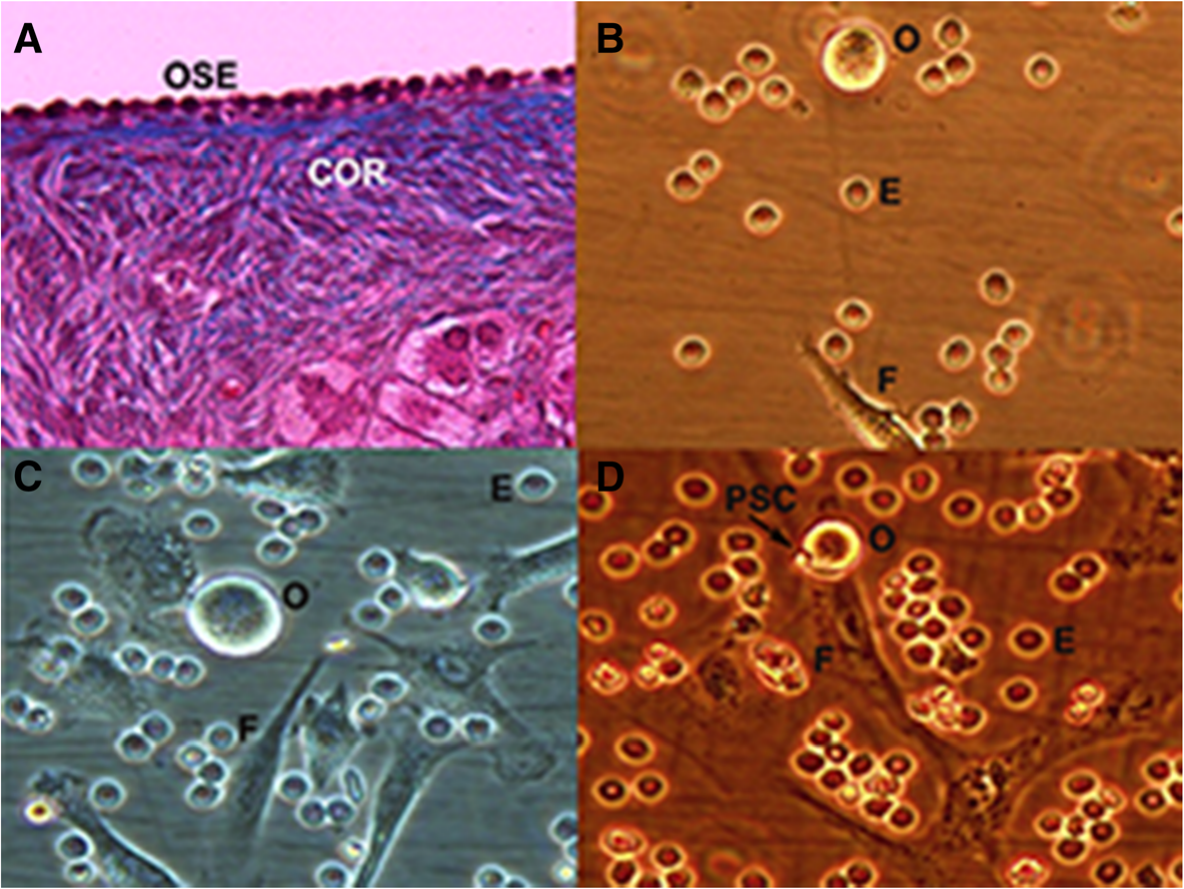
**Ovarian surface epithelium (OSE) histology and oocyte-like cells developing among putative stem cells in the OSE culture.** (**A**) OSE layer with no follicles and oocytes in the ovarian cortex (COR) after Masson’s trichrome staining of the ovarian section (light microscope, magnification 40×). (**B**, **C**) Different types of cells including erythrocyte (**E**), fibroblast (**F**), oocyte-like cells ((**O**), 10 – 15 μm) (inverted microscope, 400). (**D**) Oocyte-like cell (10 μm) developing in close contact with putative stem cells (PSC) and fibroblasts on day 7 of culture (inverted microscope, 400).

Recently, we have cultured the ovarian surface epithelium of postmenopausal women and POF patients whom their OSE layer was confirmed under inverted microscope, 400 (unpublished data). On the day 7 of the OSE cell culture, oocyte-like cells with a diameter of approximately 10–15 μm were developed among proliferating small cells—putative stem cells. (Figure 1A - D). The developed oocyte-like cells, resembling a small bubble-like structure, were developed from putative stem cells, and mostly grew attached to the fibroblasts. On the other hand, we noted that these cells did not express any germ line cell markers in the immunohistochemical examination.

### Production of offspring from OSE cells

The observations of some OSE cells transition into oocyte like cells have raised a question whether such potential can also be used to produce offspring from OSE cells. The initial study by Bukovsky et al. demonstrated that some oocyte like cells derived from OSE cells differentiated de novo into parthenogenetic blastocyst and embryo-like structures, which expressed DAZL [30,51]. These observations supported by Virant-Klun et al. showed that under certain conditions, *in vitro* isolated cells from OSE *per se* had the ability to produce oocytes or oocyte-like cells, which also could undergo parthenogenetic development to generate blastocyst-like structures [38,39]. Interestingly, Niikura et al. demonstrated high expression of the premeiotic marker Stra8 and Daz1 in the ovary of aged female mice, despite the absence of oocytes [44]. Following grafting ovarian tissue harvested from aged female germline specific green fluorescent protein (GFP) expressing mice (PE-Oct4-Gfp or TgOG2 transgenic) into the ovarian bursal sac of young adult-type female recipients, a small number of GFP-positive germ cells, as immature follicles and co-expressing the primordial oocyte marker NOBOX, were detected. However, the number of immature follicles in the ovary of the young adult mouse, exposed directly to an aged systemic environment, was decreased. These observations support the concept of rapid decline in the number of follicles being mainly due to decreased oocyte renewal rather than to an accelerated loss. In line with the previous findings, Zou et al. established a neonatal female germline stem cell (FGSC) line positive for mouse vasa homologue protein (MVH) from 5 day old and adult C57BL/6 wild-type and C57BL/6CD-1 F1 hybrid mice [9,45]. Following *in vitro* culture, retroviral infection with GFP and bromodeoxyuridine labeling FGSCs were transplanted into ovaries of chemically sterilized six-week-old C57BL/6CD-1 F1 hybrid females. Transplanted cells underwent oogenesis demonstrated high telomerase activity and the mice delivered offspring carrying the GFP gene. Recently Pacchiarotti et al. further illustrated the validation of cell lines as ovarian germ line stem cells [52]. They reported the isolation of cells containing the putative stem cells from the OSE of neonatal OG2 mice. Following aggregation with granulosa cells, formation of “embryonic bodies” containing differentiated derivatives of the three germ layers and production of early stage oocytes were demonstrated during culture.

A recent intriguing study using an antibody against DEAD [Asp-Glu-Ala-Asp] box polypeptide 4 (DDX4) coupled with a fluorescence-activated cell sorting (FACS) reported the presence of purified rare mitotically active cells with germ line specific gene expression features in both mouse and human ovaries [47]. These cells were able to spontaneously form oocytes with an approximate diameter of 35 – 50 micron. Presence of oocyte specific markers was confirmed. Further germ cell specific proteins (DNA recombinase dosage suppressor of mck1 homolog (DMC1) and synaptonemal complex protein 3 (SYCP3)) necessary for progression of meiotic recombination were identified in these cells, indicating entry to meiotic division. Chromosomal DNA content analysis revealed diploid (2n) and haploid (1n) cell populations in the *in vitro* human OSC cultures. Injection of GFP transfected human OSCs into the human ovarian cortical tissue strips resulted in generation of GFP expressing follicles 1 – 2 weeks after xenografting these strips into non-obese diabetic-severe combined immune-deficient mice. Eventually, the authors have successfully purified rare cell surface DDX4 positive oogonial stem cells from cortical tissue of reproductive aged adult human ovaries. These cells were able to develop and demonstrate both mitotic and meiotic activity. They produced oocytes *in vitro*, and more importantly *in vivo* when injected into cortical tissue strips from human ovaries.

Similar perspective has also been proposed by other group [48]. In combination of female PGCs with embryonic gonadal somatic cells were transplanted under ovarian bursa or kidney capsules of recipient mice *in vitro* for oogenesis. After epithelial stem cells (ESCs) were induced to transform into primordial germ cell-like cells (PGCLCs), histological sections revealed that PGCLCs contribute to oocyte-like cells in reconstituted ovaries *in vitro* and further matured into fully functional germinal vesicle stage, including multiple layers of granulosa and theca cells thereafter transplantation *in vivo*[48]. Combined with previous studies, the observations of this study further demonstrated that it is possible to reconstitute female germ line development *in vitro* in mice as well as other mammalian species including humans.

## Conclusion

The function of OSE during postnatal period in mammalian species remains elusive. The questions of whether germ line stem cells exist in the surface epithelium of the adult human ovaries and if they do exist, whether they can generate oocytes need to be precisely addressed. However, in the concept of reproductive medicine, infertile patients with POF, poor responders, women undergoing gonadotoxic treatment before completion of childbearing, or those young women with isolated high serum FSH levels are of interest; thus, there is no doubt that any potential approach to reform their oocytes would be of great improvement. OSE has been indicated to be a niche of oocytes during the embryonic life in human beings [8,53]. Accumulating data are present regarding to increasing evidence of the existence of putative GSC within adult mammalian OSE. A small number of PGCs/oogonia or PGC-derived VSELs with GSC characteristics can be possibly retained in postnatal and adult ovary, and under certain conditions, they may resume mitosis, enter meiosis and form new oocytes. Therefore, the presence of ovarian stem cells in the OSE of women with ovarian failure or diminished ovarian reserve are an essential concept to be investigated further in terms of their possible regenerative capacity in adult mammalians. *In vitro* OSE cultures would be beneficial models to put forth the molecular and cellular functions of OSE in entire women population including fertile and infertile. In general agreement, the presence of ovarian stem cells in the OSE appears to be evident. Considering these striking observations reported by aforementioned studies, OvaScience conducts a Phase I study in which mitochondria of oocytes will be isolated from OSCs of a woman undergoing to IVF and then, these isolated cells will be autologously injected into the oocytes of the same patient [54]. If the researchers achieve the expected results of that trial, compromising increased egg viability, embryo development and success rate of IVF, the evidence will provide an essential prospect for the management of infertile women who have been seeking for a child for years.

## Abbreviations

BM: Bone marrow; BMT: Bone marrow transplant; C-kit: Tyrosine kinase receptor for stem cell factor; DAZL: Deleted azoospermia-like; DDX4: DEAD [Asp-Glu-Ala-Asp] box polypeptide 4; DMC1: DNA recombinase dosage suppressor of mck1 homolog; ESCs: Epithelial stem cells; FGSC: Female germline stem cell; FACS: Fluorescence-activated cell sorting; GCS: Germ line stem cells; GDF3: Growth differentiation factor-3; GFP: Green fluorescent protein; KLF4: Krueppel-like factor 4; Mage-A4: Melanoma antigen-4; MVH: Mouse vasa homologue protein; NANOG: Homeobox gene transcription factor; NOBOX: Newborn ovary homeobox; OCT4: Octamer binding transcription factor 4; OSE: Ovarian stem cell; POF: Premature ovarian failure; PGCs: Primordial germ cells; SOX2: Sex determining region Y box 2; SSEA4: Stage-specific embryonic antigen-4; SYCP3: Synaptonemal complex protein 3; TgOG2: Transgenic GFP-expressing mice; VSELs: Very small embryonic-like cell(s).

## Competing interests

The authors declare that they have no competing interests.

## Authors’ contributions

OC: 1) has made substantial contributions to conception and design, 2) has been involved in drafting the manuscript, and 3) have given final approval of the version to be published. EC: 1) has made substantial contributions to conception and design, 2) has been involved in drafting the manuscript or revising it critically for important intellectual content. IT: 1) has made substantial contributions to conception and design, 2) revising it critically for important intellectual content. EY: revising it critically for important intellectual content. YS: revising it critically for important intellectual content. BT: revising it critically for important intellectual content. All authors read and approved the final manuscript.
